# Autism: A model of neurodevelopmental diversity informed by genomics

**DOI:** 10.3389/fpsyt.2022.981691

**Published:** 2022-09-02

**Authors:** Samuel J. R. A. Chawner, Michael J. Owen

**Affiliations:** MRC Centre for Neuropsychiatric Genetics and Genomics, Cardiff University, Cardiff, United Kingdom

**Keywords:** autism, genomics, co-occurring disorders, neurodiversity, neurodevelopmental outcome

## Abstract

Definitions of autism are constantly in flux and the validity and utility of diagnostic criteria remain hotly debated. The boundaries of autism are unclear and there is considerable heterogeneity within autistic individuals. Autistic individuals experience a range of co-occurring conditions notably including other childhood onset neurodevelopmental conditions such as intellectual disability, epilepsy and ADHD, but also other neuropsychiatric conditions. Recently, the neurodiversity movement has challenged the conception of autism as a medical syndrome defined by functional deficits. Whereas others have argued that autistic individuals with the highest support needs, including those with intellectual disability and limited functional communication, are better represented by a medical model. Genomic research indicates that, rather than being a circumscribed biological entity, autism can be understood in relation to two continua. On the one hand, it can be conceived as lying on a continuum of population variation in social and adaptive functioning traits, reflecting in large part the combination of multiple alleles of small effect. On the other, it can be viewed as lying on a broader neurodevelopmental continuum whereby rare genetic mutations and environmental risk factors impact the developing brain, resulting in a diverse spectrum of outcomes including childhood-onset neurodevelopmental conditions as well as adult-onset psychiatric conditions such as schizophrenia. This model helps us understand heterogeneity within autism and to reconcile the view that autism is a part of natural variability, as advocated by the neurodiversity movement, with the presence of co-occurring disabilities and impairments of function in some autistic individuals.

## The shifting sands of autism diagnosis

The diagnostic features of autism have been in constant flux since early descriptions by Sukhareva (1, 2) and later Kanner (3). Definitions have been altered six times (2) across the history of DSM and ICD (4), reflecting ongoing debates about the essential characteristics of autism and how it should be diagnosed (2). The following is not an exhaustive summary, but highlights some of the important changes in diagnostic criteria and definitions. Currently, in DSM-5, autism is defined by two key domains; atypical social communication and interaction; and restricted, repetitive behavior and interests (5). Prior to DSM-5, these two domains were conceptualized as a triad of impairments by Wing and Gould (6), with social function and communication being considered separately. Moreover, in DSM-5, Asperger syndrome and autism spectrum disorder were subsumed into a single category of autism. Previously, a diagnosis of autism spectrum disorder required delays in language development to be present in addition to social, communication and repetitive behaviors, whereas developmental delays were required to be absent for a diagnosis of Asperger syndrome.

These changes have resulted in the DSM-5 definition of autism being more inclusive, with greater phenomenological heterogeneity (2). In response to this, terminology outside the DSM-5 diagnostic framework has been developed, to delineate autism subgroups. For instance, The Lancet Commission recently introduced the term “profound autism” to indicate autistic individuals who have higher support needs (7). An important shift in our perspective of autism has come from the neurodiversity movement, pioneered by autistic activists. Neurodiversity challenges the conception of autism as a medical syndrome defined by functional deficits. Under neurodiversity, autism is seen as one form of variation within a diversity of minds (8–10). This has the potential to radically change how autism is researched and how autistic people are valued and supported (9). However not all people with autism and stakeholders identify with the neurodiversity movement (11), and concerns remain about how autistic individuals with the highest support needs, including those with intellectual disability and limited functional communication, are represented in a non-medical model (7).

These shifts in diagnosis and conceptualization have caused debate, but also reflect the inherent phenomenological basis of diagnosis. Autism is still diagnosed based on observation and reported behavior in relation to societal norms. However, if we are to move beyond behavioral definitions, there is a need for new perspectives, and, in this article, we discuss the insights genomics has provided to our understanding of autism as a diagnostic entity.

## Genetic epidemiology

Genetic epidemiological studies have shown that genetics plays a major role in the etiology of autism and have yielded high heritability estimates. Interest in the genetics of autism was initiated by a small twin study, published in 1977, which included 10 dizygotic (DZ) and 11 monozygotic (MZ) pairs and found that four out of the 11 MZ pairs (36%) but none of the DZ pairs were concordant for autism (12). A subsequent meta-analysis of seven primary twin studies yielded heritability estimates ranging from 64 to 93% (13). The emerging data from early twin studies provided important evidence challenging stigmatizing theories that autism is caused by maternal coldness or emotionless parenting styles (14). The role of maternal warmth and attachment in the etiology of autism was first proposed by Kanner and then popularized by Bruno Bettleheim’s book—The Empty Fortress (1967), which introduced and promoted the “refrigerator mother hypothesis” (15) of autism, which although now largely rejected was influential in its time.

Alongside twin studies, family studies also highlighted the high heritability of autism, indicating that the probability of a child having autism corresponds to their degree of relatedness to autistic relatives (16–19). Family studies also found that relatives of autistic individuals were more likely to exhibit behaviors consistent with a “broader autism phenotype”—consisting of sub-threshold difficulties with social skills and communication, and the presence of autistic-like personality features (20). Whereas the presence of broader autism features in parents had often been interpreted as being causative of childhood autism in line with the refrigerator hypothesis (21), the application of genetic study designs provided an important lesson that genetic correlation might underlie the relationship between parental and childhood behavior. Increased broader autism-related strengths have also been reported in the relatives of autistic individuals; many autistic individuals evince superior folk physics ability (the ability to spontaneously perceive the workings of the physical world), and fathers and grandfathers of autistic children have been found to be more than twice than likely to work in the field of engineering (22). A study of undergraduate students of physics, engineering and mathematics found they were more likely to have an autistic relative than undergraduate students studying arts subjects (23). These findings are a reminder of the potential evolutionary benefit of autistic traits in the population.

Research into the priorities of the autistic community has identified co-occurring neurodevelopmental and mental health conditions as key issues impacting wellbeing in autistic individuals (7, 24). A meta-analysis incorporating clinical, population and registry based cohorts found increased prevalence of psychiatric conditions in autistic individuals; 28% for attention-deficit hyperactivity disorder (ADHD); 20% for anxiety disorders; 13% for sleep–wake disorders; 12% for disruptive, impulse-control, and conduct disorders; 11% for depressive disorders; 9% for obsessive-compulsive disorder; 5% for bipolar disorders; and 4% for schizophrenia spectrum disorders (25). Further studies highlight increased prevalence of intellectual disability (ID) (26) and eating disorders (27). For some co-occurring conditions, the stigma faced by autistic individuals in society is likely to be an important contributing factor, but twin studies have also indicated a substantial genetic overlap between autistic traits and symptoms of other psychiatric conditions, including ID (28), ADHD (29), anxiety (30), and psychotic experiences (31). Studies of relatives of autistic individuals also find increased prevalence of co-occurring neurodevelopmental and mental health conditions (32).

## Genomics

Genomics allows genetic risk factors to be identified and measured at the molecular level of DNA variation. Its reach is limited by the technologies that can currently be feasibly applied to large samples. Most of the informative data on autism obtained to date come from genome-wide association studies (GWAS), which use genotyping arrays to identify common (>1%) single nucleotide polymorphisms (SNPs) that typically have small effects on individual risk, and rare copy number variants (CNVs), which are large deletions and duplications of DNA typically affecting multiple genes. Sequencing studies have been used successfully to detect rare single nucleotide variants (SNVs) that have large effects on individual risk and, for reasons of cost, to date most have been based on whole exome, rather than whole genome, sequencing. The identification of rare high-risk SNVs, as well as small structural variants and other mutation classes, outside of genes, and rare mutations that have small effects on risk will require whole genome sequencing in large samples (33). However, while as a consequence much genetic risk remains unaccounted for at the DNA level (34), genomic studies have yielded findings with important implications for our understanding of autism as a biological entity.

Genomic studies have revealed that autism has a complex polygenic architecture, involving risk alleles across the frequency spectrum (16). In other words, an individual’s genetic risk of developing autism is determined by a constellation of genetic risk factors some of which are rare and some common in the general population. Approximately 4–5% of individuals with autism have a recognized syndrome consisting of a clinically defined pattern of somatic abnormalities and a neurobehavioral phenotype which may include autism (35). Most of these are associated with a known genetic cause, often rare mutations or CNVs, and examples include tuberous sclerosis and fragile X syndrome. Recent genomic research has focused on large samples of individuals with autism, the great majority of whom do not have syndromic autism. This has identified rare SNVs in over 100 genes that confer large effects on individual risk (36). These mutations are defined as “damaging” in the biological sense that that they disrupt protein quantity or structure, and they tend to be found in genes that are “constrained” in that they rarely contain damaging mutations in the general population. They also frequently, but not exclusively, occur *de novo*, i.e., as new mutations not present in either parent. Large, rare CNVs are also associated with a high risk of autism and occur in 4–10% of autistic individuals (37–39). These are also frequently *de novo* but can be transmitted from affected or unaffected parents and found in unaffected relatives.

Although rare risk alleles confer large effects on individual risk, it appears that the great majority of the identified genetic risk at a population level is conferred by the *en masse* effects of a very large number, probably thousands, of common risk alleles each of which has a very small effect on individual risk (40). It also seems that in those with rare mutations, the burden of common risk alleles combines additively with the risk conferred by the rare mutations to determine individual risk (34, 41).

As well as beginning to reveal, in broad terms, the genetic architecture of autism, genomic findings also help us understand the possible relationships between autism and other conditions and traits by revealing a lack of specificity of genetic risk to autism. Notably, genetic variants associated with autism also increase risk for conditions that frequently co-occur in autistic people and to which their relatives are at increased risk. Thus, common variant genetic risk is at least modestly correlated with that for other neurodevelopmental and psychiatric conditions such as ADHD, depression and schizophrenia (40). Moreover, rare risk variants, both SNVs and CNVs, overlap with those that confer risk to other childhood neurodevelopmental conditions such as ID, ADHD, as well as schizophrenia, a neurodevelopmental condition that typically has its onset in adolescence or early adulthood (42, 43).

**FIGURE 1:**
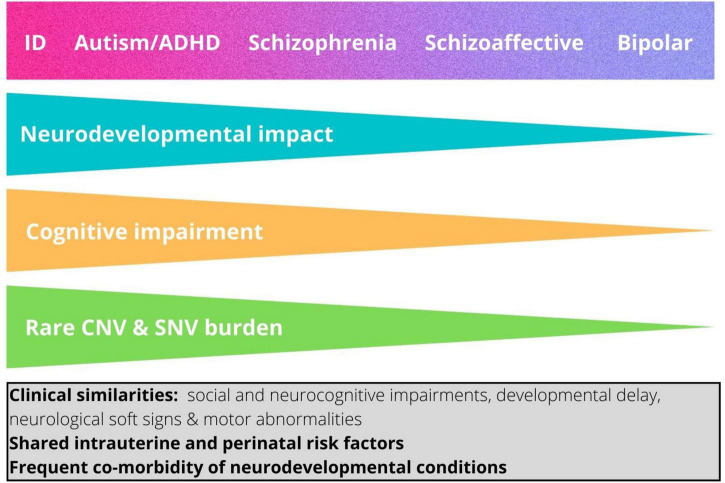
The neurodevelopmental continuum. This shows the hypothesized relationship between magnitude of neurodevelopmental impact and categorical neurodevelopmental and psychiatric diagnoses (42). The relative impact of copy number variants and damaging point mutations and the degree of associated cognitive impairment typically associated with each diagnosis are also shown. ID, intellectual disability; ADHD, attention-deficit/hyperactivity disorder. The box shows features that are shared by the different neurodevelopmental diagnostic categories.

Interestingly, the enrichment of rare risk mutations is not equal across neurodevelopmental conditions, but is greatest in ID, followed respectively by autism, ADHD, and schizophrenia (42). These findings suggest that neurodevelopmental conditions, including autism, rather than being etiologically discrete entities, are better conceptualized as lying on a neurodevelopmental continuum, with the major clinical conditions reflecting in part the magnitude of the impact on brain development and resulting functional outcomes (42, 44). Thus, within this continuum, neurodevelopmental conditions occupy a gradient of decreasing neurodevelopmental impact as follows: ID, autism, ADHD, schizophrenia (42) (Figure 1).

**FIGURE 2:**
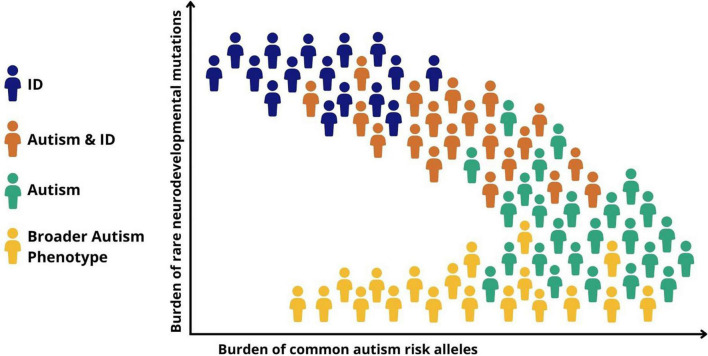
Genomic and symptomatic heterogeneity in autism. Simplified schematic representation of the relationship between different classes of genetic risk factors and neurodevelopmental outcomes. ID, intellectual disability; Autism + ID, autism and co-occurring ID; Autism, childhood autism with moderate language delays; Broader autism phenotype, variation in social behavior and adaptive functioning seen in the general population.

Recent genomic data suggest that the notion of a neurodevelopmental continuum can also be extended to help understand heterogeneity and the large variability in cognitive and functional ability within autism. Autistic children with high support needs and particularly those with co-occurring ID are more likely to have rare risk mutations, particularly those that have occurred *de novo*, compared to autistic individuals without ID (45). This is congruent with a gradient of decreasing neurodevelopmental impact from autism and co-occurring ID, through childhood autism with moderate language delays, to autism without ID or language delays (Figure 2). Common variants, on the other hand, appear to play a relatively larger role in autistic individuals without ID (40, 46). This helps to explain why autism without co-occurring ID is more heritable than autism with co-occurring ID, which has a prominent contribution from *de novo* rare mutations, which are, by definition, not carried by parents.

As we have seen, family studies suggest that there may be a genetic relationship between diagnosed autism and autistic traits in the general population. This has been confirmed by genomic studies showing that genetic risk for autistic traits varies across the population with contributions from both common and rare risk variants, with those carrying a greater burden of risk alleles being more likely to meet diagnostic criteria (44, 47). As well as suggesting a continuous risk landscape among neurodevelopmental conditions including autism, genomic findings also point to a second continuum of genetic risk between autism and typical variation in social behavior and adaptive functioning (communication and daily living skills) seen in the population (Figure 2). Regarding the genetic etiology of repetitive and restricted behaviors (RRB) in the population, a twin study has demonstrated high heritability for RRBs, but that RRBs have low genetic covariation with social traits, indicating potentially different genetic etiologies for different autism domains (48).

## Autism as a diagnostic entity

How do genetic findings inform our understanding of autism as a diagnostic entity? There is strong evidence that susceptibility has a genetic, and therefore a biological basis, but genetics does not support the notion that autism is a biological entity that is distinct from other clinical conditions or neurotypical variation. Instead, the data suggest that autism can best be understood in relation its position in two continua. On the one hand, it can be conceived as lying at one end of continuous population variation in social and adaptive functioning, underpinned by a combination of multiple alleles of small effect. On the other, it can be seen as part of a broader neurodevelopmental continuum whereby rare, frequently *de novo*, genetic mutations that confer high individual risk impact the developing brain, resulting in a spectrum of outcomes including other childhood-onset neurodevelopmental conditions such as ID and ADHD as well as adult-onset psychiatric conditions such as schizophrenia and bipolar disorder (42) (Figure 1). We do not propose that these two continua underlie two distinct types of autism, rather they represent biological dimensions that combine to different extents in autistic individuals. Indeed, recent research has highlighted the importance of the combined effects of common polygenic variability and rare variants in conferring risk to autism (34, 41, 46).

Genetic findings also shine a light on heterogeneity within autism. Although the evidence does not support the existence of a simple dichotomy between the effects of rare and common genetic variation, it seems that *de novo* rare high-risk mutations play a relatively greater role in more severely impaired cases such as those with childhood onset autism or autism and co-occurring ID, whereas less impaired individuals reflect a greater contribution from common genetic variants that underlie variation in autistic traits in the population (Figure 2) (46). It is important to stress that these genetic mechanisms are not discrete, with rare and common risk variants combining to determine both an individual’s risk of autism and whether, and if so to what extent, co-occurring disabilities and impairments of function might be present (34).

The overlap between neurodevelopmental conditions indicates that there are likely to be biological dimensions that transcend current diagnoses, and these may provide a more useful system of characterizing neurodevelopmental diversity. Indeed, this mirrors findings from neuroimaging and neuropsychology studies of neurodivergent individuals which have identified a range of transdiagnostic dimensions, examples include global measures of brain connectivity, hyperactivity and impulsivity, inattention, social communication, executive functioning, and phonological processing (49, 50).

## Implications

Our model helps us understand how to reconcile that autism is a part of the natural variability within human brains and minds as advocated by the neurodiversity movement, with the fact that disability is a reality for some autistic people and their families (7). It provides a basis for the idea that a medical model may be appropriate in some instances, where needs are high, alongside a social model of understanding and supporting autistic individuals.

Genomic research does not indicate that autism is a discrete biological entity. Rather it supports a dimensional approach both to heterogeneity within autism and to the relationship between autism and other neurodevelopmental and psychiatric conditions. This in turn supports calls for transdiagnostic approaches to both research and clinical practice (50). Ill-fitting diagnostic criteria will impede progress toward identifying the barriers that neurodivergent individuals encounter, understanding underpinning mechanisms and finding the best route to supporting them (50). Current diagnostic categories fail to capture the extensive symptom heterogeneity within categories, or to accommodate the extensive overlap across supposedly distinct diagnostic entities. Current diagnoses also fail to capture the needs of many children who require additional support in the broad areas of learning, behavior or social functioning, and many children whose symptoms do not reach arbitrary thresholds but who nevertheless have significant difficulties cannot access support or care.

## Prospects

Autism genomics is still at an early stage and much genetic risk remains unaccounted for at the DNA level. We can expect to learn a great deal more from the application of new and emerging approaches (16) that will refine our approach to diagnosis, illuminate the underlying biology, identify novel treatment and early intervention targets for co-occurring conditions. However, genomics is already changing the lives of some families with an autistic child. Children with signs of early neurodevelopmental delay are increasingly being referred for genetic testing within clinical services to detect rare variants (51). For many families a genetic diagnosis can be the end of a diagnostic odyssey and can help explain the presence of co-occurring conditions, which can then inform tailored clinical care (51). A study of a US healthcare service that screened adults for rare neurodevelopmental CNVs explored the reactions of adults receiving a genetic diagnosis. 95% of these were positive or neutral and many individuals experienced emotionally poignant responses to learning a medical reason for lifelong cognitive and psychiatric disabilities (52). However important ethical concerns have been raised by the autistic community concerning the potential misuse of genetic research findings for eugenics (53). It is therefore important that genetic research is coproduced with the autistic community and stakeholders, and that data sharing from genetic studies is regulated appropriately. Working in partnership with the autistic community on identifying which aspects of their healthcare can most benefit from genomic insights will be crucial to ensuring success.

## Author contributions

SJRAC and MJO wrote the manuscript. All authors contributed to manuscript revision and approved the submitted version.
